# Measuring body temperature of freely moving mice under an optogenetics-induced long-term hypothermic state

**DOI:** 10.1016/j.xpro.2023.102321

**Published:** 2023-05-31

**Authors:** Tohru M. Takahashi, Takeshi Sakurai, Arisa Hirano

**Affiliations:** 1 Institute of Medicine, University of Tsukuba, Tsukuba, Ibaraki 305-8575, Japan; 2 International Integrative Institute for Sleep medicine (WPI-IIIS), University of Tsukuba, Tsukuba, Ibaraki 305-8575, Japan

**Keywords:** Neuroscience, Behavior

## Abstract

We present a protocol for inducing a hibernation-like state in free-moving mice using optogenetics. We have recently developed an optogenetic technique utilizing modified *Opsin4*, which is activated by weak blue light, resulting in prolonged neuronal excitation. We describe a protocol that includes detailed instructions for virus injection, implantation of optic fibers and temperature transmitters, photostimulation, and real-time recording of body temperature in mice. This method is valuable for investigating the mechanisms underlying torpor and thermoregulation in mice.

For complete details on the use and execution of this protocol, please refer to Takahashi et al.^1^

## Highlights

• Two-way body temperature recording during optogenetics induction of hypothermia
• Setting up the thermographic camera and telemetry system
• AAV transduction and optic fiber insertion for excitatory manipulation of Q neurons
• Transmitter implantation in abdominal cavity for core body temperature monitoring

## Abstract (editor highlights)

Publisher’s note: Undertaking any experimental protocol requires adherence to local institutional guidelines for laboratory safety and ethics.

## Graphical abstract

**Figure:**
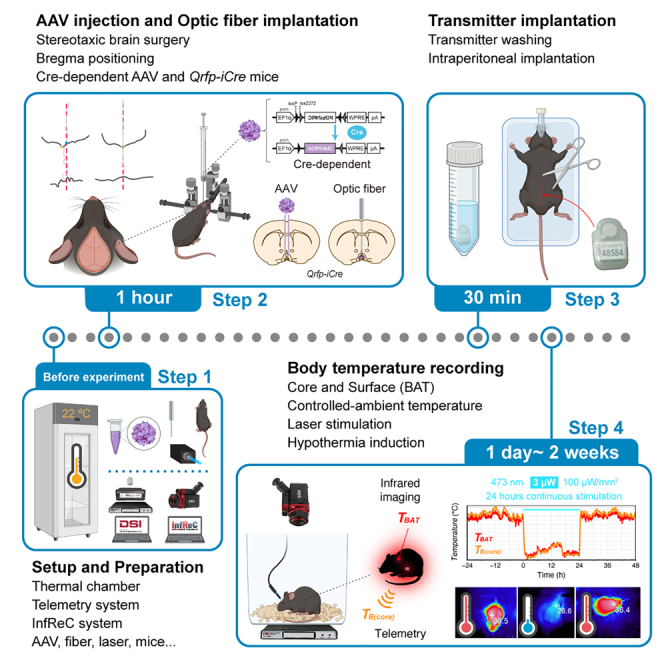

## Before you begin

We have previously reported a hibernation-like state induced by the chemogenetic activation of Quiescence-inducing neurons (Q neurons) in mice.^2^ Q neurons are localized in the anteroventral periventricular nucleus (AVPe) of the hypothalamus and are molecularly defined by the expression of the neuropeptide pyroglutamylated RFamide peptide (QRFP). The Q-neuron-induced hypothermic and hypometabolic state (QIH) is similar to the hibernation observed in hibernators, such as ground squirrels and Syrian hamsters. We recently developed an optogenetic tool using a blue-light photoreceptor, human Opsin4 (OPN4), to generate a long-lasting QIH with a higher temporal resolution than the chemogenetics, a stimulatory hM3Dq designer receptor exclusively activated by the designer drug (DREADD) system.^1^ Modified OPN4 (OPN4dC) lacking the C-terminal region can be activated by weak light stimulation, allowing for prolonged manipulation of Q neurons (at least 24 h) without any brain damage. Here, we describe the materials and methods used for optogenetically inducing QIH and monitoring the body temperature of freely-moving mice. This technique will be useful in understanding the neuronal and molecular mechanisms of thermoregulation and hibernation. Additionally, OPN4dC optogenetics, which activates Gq signaling, can be used to manipulate other types of neurons and non-neuronal cells.^1^

Several studies have reported the induction of hypothermia in mice by neuronal manipulation and the measurement of body temperature during the process. However, the duration of optogenetic manipulation in these studies was limited to a few hours (Table 1). On the other hand, chemogenetics has been mainly used to induce hypothermia for a longer period (beyond a day),^3^^,^^4^^,^^5^ where the endpoint of stimulation is unclear owing to the gradual disappearance of the drug effect. Our previous study made long-term (at least 24 h) neuronal stimulation possible even with optogenetics at a higher time resolution.^1^ Notably, OPN4dC does not require high-power light, which could artificially increase the temperature in tissue.^6^ Since the preoptic area (POA) contains warm-sensitive neurons, which endogenously decrease the body temperature when activated,^7^^,^^8^ stimulating light for optogenetics needs to be weak enough not to produce heat by light-illumination itself, leading to activation of these warm-sensitive neurons.

**Table 1:** Literature using excitatory optogenetics to induce a hypothermic state in mice

References	Core/ BAT	Opsin	Time scale	Temp. drop	Mouse line	Brain region
Tan et al.^9^	BAT	SSFO	30 min	3°C 2°C	Adcyap1-Cre BDNF-Cre	VMPO
Zhao et al.^10^	Core	ChR2	1 h	3°C	Vgat-Cre	vLPO
Abbott et al.^11^	Core	ChR2	20 min	5°C	Vglut2-Cre	MnPO
Takahashi et al.^2^	BAT	SSFO	2 h	13°C 10°C 2°C	Qrfp-iCre	AVPe AVPe→DMH AVPe→RPa
Piñol et al.^12^	Core	ChR2	20 min	2°C	Wild type	POA
Yamagata et al.^13^	BAT (skin)	ChR2	1 h	4°C	GAD2-Cre	LPO
Qian et al.^14^	Both	ChR2	1 h	6°C	Vglut2-Cre	apMPOA
Takahashi et al.^1^	Both	OPN4dC SSFO ChR2	24 h 3 h 12 h	10°C 12°C 12°C	Qrfp-iCre	AVPe
Padilla et al.^15^	Both	ChR2	2 h	2°C	Kiss1-Cre	ARH→POA
Jeong et al.^16^	Both	ChR2	1 h	1–2°C	ChAT-Cre	DMH
Liu et al.^17^	BAT	ChR2	15 min	3°C	Vglut2-Cre	PSTh→NTS
Yang et al.^18^	Both	ChIEF ChlEF ChlEF	30 min	2°C 4°C 2°C	Vglut2-Cre Pdyn-Cre CCK-Cre	LPB→VMPO
Norris et al.^19^	Both	ChR2	15 min	5°C 3°C 4°C	Pdyn-Cre Penk-Cre Vglut2-Cre	PBN→POA
Schneeberger et al.^20^	BAT	ChR2	20 min	1°C	Vgat-Cre	DRN→RPa

To monitor the body temperature of the mice, we describe two methods: the use of implanted temperature sensors and infrared thermographic cameras. The former method allows for monitoring of the mouse core body temperature (T_B_), whereas the latter enables monitoring of the body temperature of the surface skin above the brown adipose tissue (T_BAT_) and tail. We verified a strong correlation between T_B_ and T_BAT_. Researchers can choose to use one or both methods, depending on the objectives of their experiments.

### Institutional permissions (if applicable)

All animal experiments described in this protocol were approved by the Animal Care and Use Committee of the University of Tsukuba and were carried out in accordance with the guidelines of the Committee.

### Preparations for viral injection and implantation of optic fibers

**Timing: 1 day**

1. Preparation of Adeno-associated virus vectors (AAV).
  a. Optogenetics using C-terminal deletion-type OPN4 (OPN4dC) is particularly suitable for the prolonged manipulation of Q neurons. The construct for the AAV vectors Cre-dependently expressing OPN4dC fused with mCherry (pAAV-EF1a-DIO-hOPN4dC-mCherry) is available upon request.
  b. Thaw the virus stock solution on ice and then store it at −80°C after being divided into aliquots. To avoid multiple freeze-thaw cycles, it is recommended to make small aliquots (3–4 μL) for single use.
2. Preparation of animals.
  a. To specifically manipulate Q neurons, it is recommended to use heterozygous *Qrfp-iCre* mice (strain name: B6J.B6N-Qrfp<tm2.1(icre)Stak> strain ID; RBRC11137). The age and sex of the mice depend on the purpose of the experiment.
3. Setting up a brain microinjection system, as shown in Figure 1 and the key resources table.
  a. Stereotaxic for mice or small animals.
  b. Small animal veterinary anesthesia machine and isoflurane.
  c. Micro syringe pump for stereotaxic brain injection.
  d. Stereotaxic holders of syringes and optic fibers.

**Figure 1:**
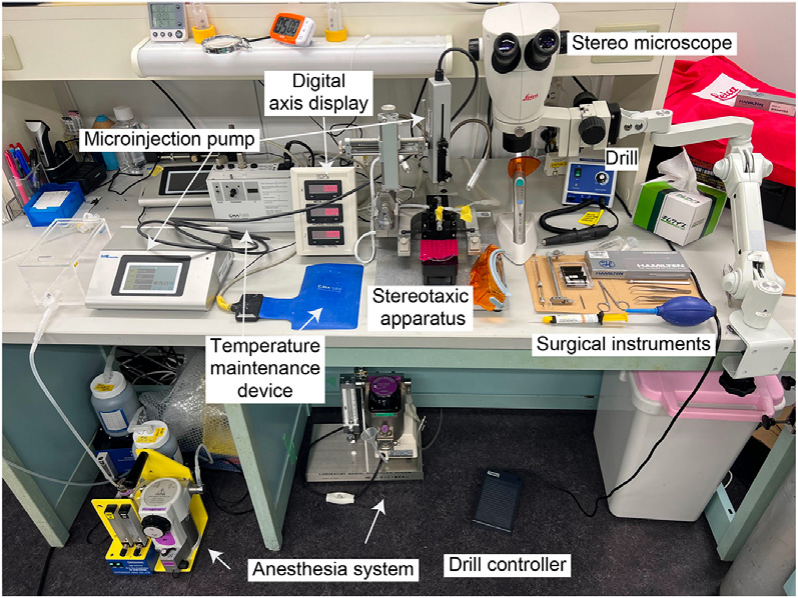
Brain stereotaxic system for AAV injection and optical fiber implantation The components of the brain stereotaxic system include stereo microscopes, an isoflurane gas anesthesia system, a digital stereotaxic instrument, a microinjection pump, a heat pad, and a skull drill.
4. Preparation of other surgical instruments and reagents, as shown in Figure 1 and the key resources table.
  a. 70% ethanol and sterilized saline.
  b. Sterilized scissors.
  c. Sterilized tweezers.
  d. Hamilton syringe (10 μL) and 33 gauge needles.
  e. Surgical cranial drill and drill bit (0.5 mm diameter).
  f. Optic fiber tips (200 μm diameter, 0.5 NA, 6 mm length).
  g. Dental cement and curing light.
  h. Heat pad.
  i. Stereoscopic microscope.

***Alternatives:*** You can also use glass micropipettes for virus injection. Glass micropipettes are more suitable for the injection of a small volume of virus vector, while the Hamilton syringe is easier to handle.

### Preparation for transmitter implantation

**Timing: 3 days**

**Figure 2:**
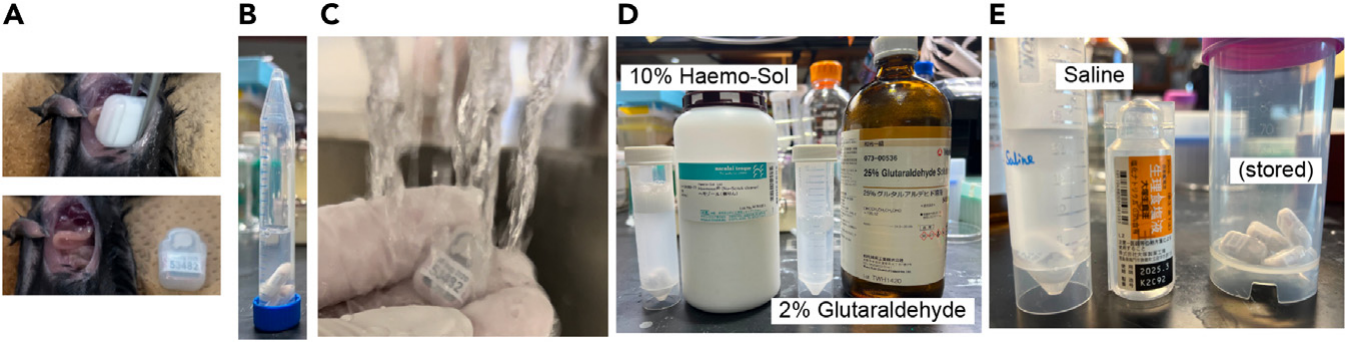
Cleaning and sterilization of transmitter (A) Used transmitter should be collected before perfusion fixation. (B) Keep transmitters in water until the cleaning to prevent them from being dried. (C) Wash off any blood or tissue stuck with running water. (D) Two chemicals used for the cleaning. (E) Transmitters can be reserved in saline or by keeping them dried at room temperature.
  c. Store TA-F10 in water until chemical washing is started (Figure 2B).
  d. Gently wash off any blood or tissue from the sensor with running water (Figure 2C).
  e. Soak TA-F10 in 10% Haemo-Sol diluted with water for at least 4 h; ideally more than 10 h (Figure 2D).
  f. Soak TA-F10 in a 2% glutaraldehyde solution diluted with water for at least 4 h; ideally more than 10 h. (Figure 2E).
  g. Soak TA-F10 in sterilized saline for at least 4 h to remove the glutaraldehyde.
    ***Note:*** After **step 5g**, the TA-F10 sensors can be kept in saline for up to 48 h prior to the implantation procedure. For long storage, dry the sensors thoroughly after cleaning and store them at room temperature (20°C–24°C, Figure 2E).

5. Preparation of temperature transmitter.
  a. The TA-F10 (Data Sciences International, USA) is an ideal device for measuring core body temperature (T_B_) and locomotor activity in small animal models, such as mice, at high resolution.
    ***Note:*** The TA-F10 can be reused unless the internal battery is exhausted. The instructions for reusing the sensors are provided below. Because they are not rechargeable, turn off the sensor after the experiment. The battery of TA-F10 can be regenerated by the manufacturer, and the cost of regeneration is lower than that of purchasing a new device.
    **CRITICAL:** After completing the measurements, it is important to thoroughly wash the TA-F10 with an enzymatic detergent and sterilize it for reuse.
  b. After completing the measurements, collect the TA-F10 from the abdominal cavity of animals prior to perfusion with paraformaldehyde (PFA) (Figure 2A).

6. Preparation of other surgical-related instruments and reagents.
  a. 70% ethanol and sterilized saline.
  b. Sterilized scissors.
  c. Sterilized tweezers and forceps.
  d. Sterilized suture needle and suture for mice.
  e. Small animal veterinary anesthesia machine and isoflurane.
  f. Heat pad.

### Preparation for body temperature recordings and analysis platform

**Timing: 1 day**

**Figure 3:**
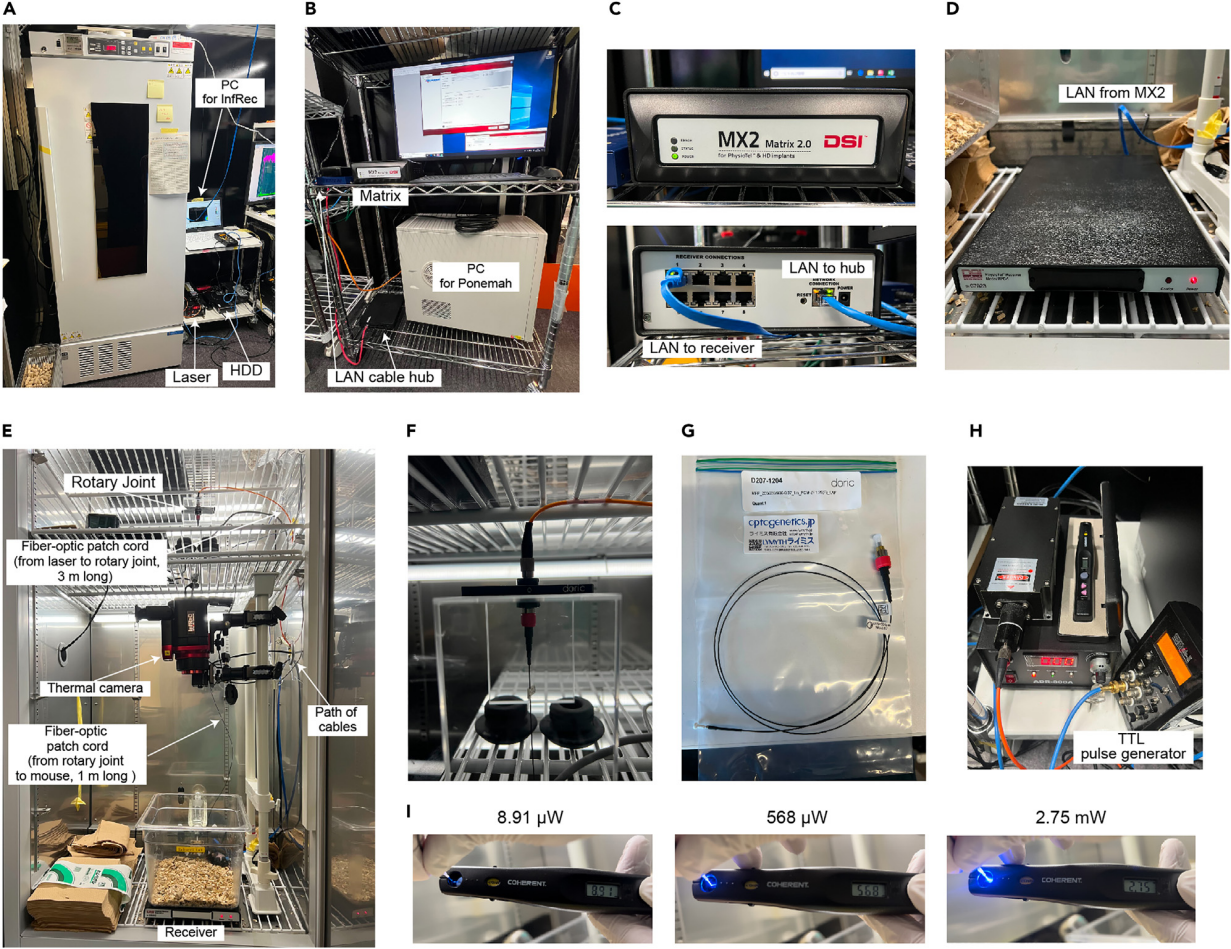
Body temperature recording platform (A) Thermostatic chamber. (B) PONEMAH telemetry instrument used to measure the core body temperature. The receiver board is connected to the Matrix with a LAN cable and placed inside the thermostatic chamber. (C) Matrix 2.0 (MX2). (D) Receiver board (RPC-1). (E) Recording environment. The receiver board is placed under the recording cage. The thermal camera is placed above the cage (about 80 cm) to record superficial body temperature. A large space above the cage and a long patch cord are recommended for long-term recording. (F) Rotary joint. (G) Fiber optic patch cord (1 m-long). (H) The laser source, laser checker, and TTL pulse generator. (I) Checking laser intensity from the fiber tip. 10 μW is sufficient for OPN4dC stimulation.
  b. A computer, in which the recording software (PONEMAH Physiology Platform v.6.30, Data Sciences International) is installed (Figure 3B).
  c. Matrix 2.0 (MX2) (Figure 3C).
  d. PhysioTel® Receiver (RPC-1) (Figure 3D).
  e. LAN cables and a hub (Figure 3C).
  f. Magnet and radio.

7. To monitor the body temperature of mice through the implanted transmitters, prepare the PhysioTel and PhysioTel HD telemetry platforms. The following hardware used in our study is also listed in the key resources table.
  a. Thermal chamber (Figure 3A).

***Alternatives:*** The thermostatic chamber (HC-100, SHIN-FACTORY) can be replaced with other chambers with similar specs. The chambers should be equipped with an air circulator and light controller.

8. To monitor the T_BAT_, prepare the infrared thermographic platform. The thermal camera should be able to detect all areas of the mouse cage floor. The following hardware used in our study is also listed in the key resources table.
  a. Thermal imaging camera (InfReC R550EX, Nippon Avionics, Figure 3E)
  b. A computer, in which the InfReC recording software (InfReC Analyzer NS9500 Professional, Nippon Avionics) is installed.
  c. Thermal chamber, which can be the same as one prepared in **step 7.**
  d. Hair clipper and hair removal cream.

***Alternatives:*** The infrared thermal camera (InfReC R550EX) can be replaced with other cameras with similar specs such as VarioCAM® HD head 800 (InfraTec).

### Preparation for optogenetic manipulation

**Timing: 1 day**

9. The following hardware and software used for optogenetic manipulation in our study are also listed in the key resources table.
  a. Fiber-optic Rotary Joints for manipulation in free-moving mice (Figure 3F).
  b. Mono fiber-optic patch cords (Figure 3G).
  c. Optic laser (Figure 3H). The wavelength of light should be ideal for activation of OPN4 (λmax = 460–480 nm). The light power at the fiber tip should be able to be reduced to 10 μW (Figure 3I).
  d. Pulse generator (Figure 3H).

**CRITICAL:** Optimize the length of the fiber-optic patch cord according to the distance between the mouse cage and the rotary joints to prevent being damaged by mice as well as to allow mice to move freely.

## Key resources table

**Table:** 

REAGENT or RESOURCE	SOURCE	IDENTIFIER
**Bacterial and virus strains**		

AAV10-EF1a-DIO-hOPN4dC-mCherry	Takahashi et al.^1^	N/A

**Chemicals, peptides, and recombinant proteins**		

Haemosol N.S	Nacalai Tesque	Cat# 04558-71
25% Glutaraldehyde solution	Fujifilm Wako Pure Chemical Corp.	Cat# 4987481269989
HIBITANE® 5% liquid	Sumitomo Pharmaceutical Co., Ltd.	Cat# 13800AZZ00776000
Vaseline	Unilever	
Isoflurane	Pfizer	Cat# 4987114133403
Ethanol (99.5)	Fujifilm Wako Pure Chemical Corp.	Cat# 057-00456
Otsuka Normal Saline	Otsuka Pharmaceutical Factory, Inc.	Cat# 035081517
Hamilton™ Needle Cleaning Concentrate 18311	Hamilton Company	Cat# 18311

**Experimental models: Organisms/strains**		

Mouse: *Qrfp-iCre* (B6J.B6N-Qrfp<tm2.1(icre)Stak) (Heterozygous; female and male mice; 8–40 weeks)	Takahashi et al.^2^	BRC# RBRC11137

**Oligonucleotides**		

PCR primer sequences for genotyping of *Qrfp-iCre* mice *Qrfp* wt foward 5′- cagtcagcagctatccctcc (361 bp) *Qrfp*-wt reverse 5′- accgtcttgcctccctagacg *Qrfp*-iCre foward 5′- gagggactacctcctgtacc (630 bp) *Qrfp*-iCre reverse 5′- tgcccagagtcatccttggc	Takahashi et al.^2^	N/A
Primer for titration of AAV vectors Forward 5′- actgtgtttgctgacgcaac Reverse 5′- agcgaaagtcccggaaag	Takahashi et al.^1^	N/A

**Recombinant DNA**		

Plasmid: pAAV-EF1a-DIO-hOPN4dC-mCherry	Takahashi et al.^1^	N/A

**Software and algorithms**		

PONEMAH Physiology Platform v.6.30	Data Sciences International.	https://www.datasci.com/
InfReC Analyzer NS9500 Professional	Nippon Avionics	https://www.avio.co.jp/
InfReC Analyzer NS9500 Standard	Nippon Avionics	https://www.avio.co.jp/

**Other**		

Hamilton Neuros Syringe, Model 1701 RN, 33 gauge, Point Style 3, 10 μL,	Hamilton Company	Cat# 65460-05
Neuros Replacement Needle, 33 gauge	Hamilton Company	Cat# 65461-01
Micro syringe pump	KD Scientific	Legato 130
Optic cannula (200-μm diameter, NA:0.50, 6.0 mm long)	Kyocera Corporation	Cat# F0617S02A4PW060
Small Animal Stereotaxics	David Kopf instruments	Model 942
Small animal veterinary anesthesia machine	Muromachi Kikai Co., Ltd.	MK-AT210D
Stereoscopic microscope	Leica Microsystems	Leica S9E
Vaseline	Kenei Phamaceutical	N/A
Surgical cranial drill	Muromachi Kikai Co., Ltd.	HSD-1000
Drill bit carbon steel burrs	Canada Fine Science Tools	Cat# 19007-05
Dental cement (RelyX™ Unicem 2 Automix Self-Adhesive Resin Cement A2)	3M	Cat# 56849
Curing light	Woodpecker	Cat# 18404003
Thermostatic chamber	SHIN-FACTORY	Cat# HC-100
Thermal-imaging camera	Nippon Avionics Co.	InfReC R550EX
Temperature transmitter (TA-F10)	Data Sciences International	Cat# TA-F10
Sterilized suture needle	Natsume Seisakusho Co., Ltd	C-24-523-R
Sterilized suture	Natsume Seisakusho Co., Ltd	C-23-B1-50
Matrix 2.0 (MX2)	Data Sciences International	Matrix 2.0 (MX2)
PhysioTel® receiver	Data Sciences International	RPC-1
462 nm blue diode laser	Shanghai-Laser	BLM462TA-200FC ADR-180A462nm
TTL pulse generator	Amuza	STOmk-2
Fiber-optic rotary joints	Doric Lenses	FRJ_1x1_FC-FC
Mono fiber-optic patch cord (laser to rotary joint)	Doric Lenses	MFP_400/440/LWMJ-0.22_3m_FC-FC
Mono fiber-optic patch cord (rotary joint to mouse)	Doric Lenses	MFP_200/230/900-0.57_1m_FCM-ZF1.25(F)_LAF
Laser checker	Coherent	Cat# 12-394

## Step-by-step method details

### AAV injection and optical fiber implantation

**Timing: 30 min**

This step describes the procedure for injecting AAV-expressing hOPN4dC-mCherry at the site of the mouse AVPe and implanting an optic fiber for laser stimulation. It is recommended to perform both AAV injection and optic fiber implantation in a single operation to avoid damage caused by a second operation and ensure accurate targeting of the AVPe.

1. Bilateral AAV microinjection into the AVPe (Figure 4).
  a. Prepare the surgical instruments (Figure 4A).
  b. Wash the Hamilton syringe (33G needle) with 70% EtOH first and then saline in this order three times each (Figure 4B).
    **CRITICAL:** Follow the order of washing because EtOH can deactivate the AAV.
  c. Withdraw AAV vectors into the syringe, which is set in the injection pump, taking care not to include bubbles inside it (Figure 4C).
  d. Anesthetize *Qrfp-iCre* heterozygous mice with ventilated 1%–2% isoflurane for 5 min (Figure 4D).
  e. Affix mice to a stereotaxic frame and turn the heat pad ON (Figure 4E).
    **CRITICAL:** It is harmful to mice if they are warmed too much by the heat pad. Set the temperature at 34°C–36°C and place a paper towel between the mouse and the pad. Troubleshooting 1.
  f. Sanitize the head with 70% EtOH, cut the skin with scissors to expose the skull and apply Vaseline to the eyes for protection.
  g. Dry the surface of the skull with a blower to make the position of bregma clear (Figure 4F).
  h. Use a small amount of hydrogen peroxide (H_2_O_2_) or 70% EtOH to remove the periosteum and clean the surface of the skull.
  i. Adjust the height of the nose clamp to set bregma and lambda at the same horizontal level.
  j. Similarly, adjust the position of either ear bar to make the brain mediolaterally horizontal.
  k. Find the bregma with a stereoscopic microscope and mark the sites of injection and implantation on the skull (coordinates relative to the bregma: +0.38 mm AP, ±0.20 mm ML for viral injection, +0.38 mm AP, 0.00 mm ML for optic cannula implantation) (Figures 4G–4I). Troubleshooting 2.
  l. Set the position of the needle tip to the bregma and move the syringe to the injection site (Figure 4J).
  m. Make a hole with a drill according to the mark (Figure 4K).
    ***Note:*** It is recommended not to break the dura when drilling.
    ***Note:*** Adding a small amount of sterile saline to the surface of the cerebral cortex at the injection site can help needles and fiber enter the brain tissue and reduce the damage to brain tissue.
  n. Confirm a flow of AAV from the needle tip before and then insert the needle into the brain (5.10 mm depth from the surface of the skull).
  o. Penetrate the dura with the needle and inject 0.15 μL AAV vector into the AVPe.
    ***Note:*** AAVs with titers of >1.0 × 10^13^ genome copies (gc)/mL usually yield good expression of opsins for optogenetics.
  p. Coordinates relative to the bregma: +0.38 mm AP, ±0.20 mm ML, −5.10 mm DV for adult mice in our study (Figure 4L)
  q. Set injection speed at 0.1 μL/min or less (Figure 4M).
    ***Note:*** There is a large individual variation in the sutures of the mouse skull, and the location of the bregma is often difficult to pinpoint (Figures 4H and 4I). Figure 4N shows an example of the bregma and two injection sites (drill marks).
  r. Wait for 5 min after the injection before removing the syringe to prevent the backflow of the injected virus.
  s. Confirm a flow of AAV from the needle tip again before the injection at the contralateral site of AVPe.
  t. Inject another 0.15 μL AAV vector into the contralateral AVPe. Troubleshooting 3
  u. Wipe the needle with 70% EtOH and paper to remove blood and tissue fluid.
  v. To prevent the needle to be clogged by blood or tissue fluid, put the needle in saline in a 1.5 mL tube and infuse a small amount of AAV from the syringe (about 2 s at the same rate as an injection to the brain).
    **CRITICAL:** When the needle is clogged by blood or tissue fluid interfering with the viral injection, replace the needle with a new one.
  w. Remove the injection pump from the stereotaxis stand.

**Figure 4:**
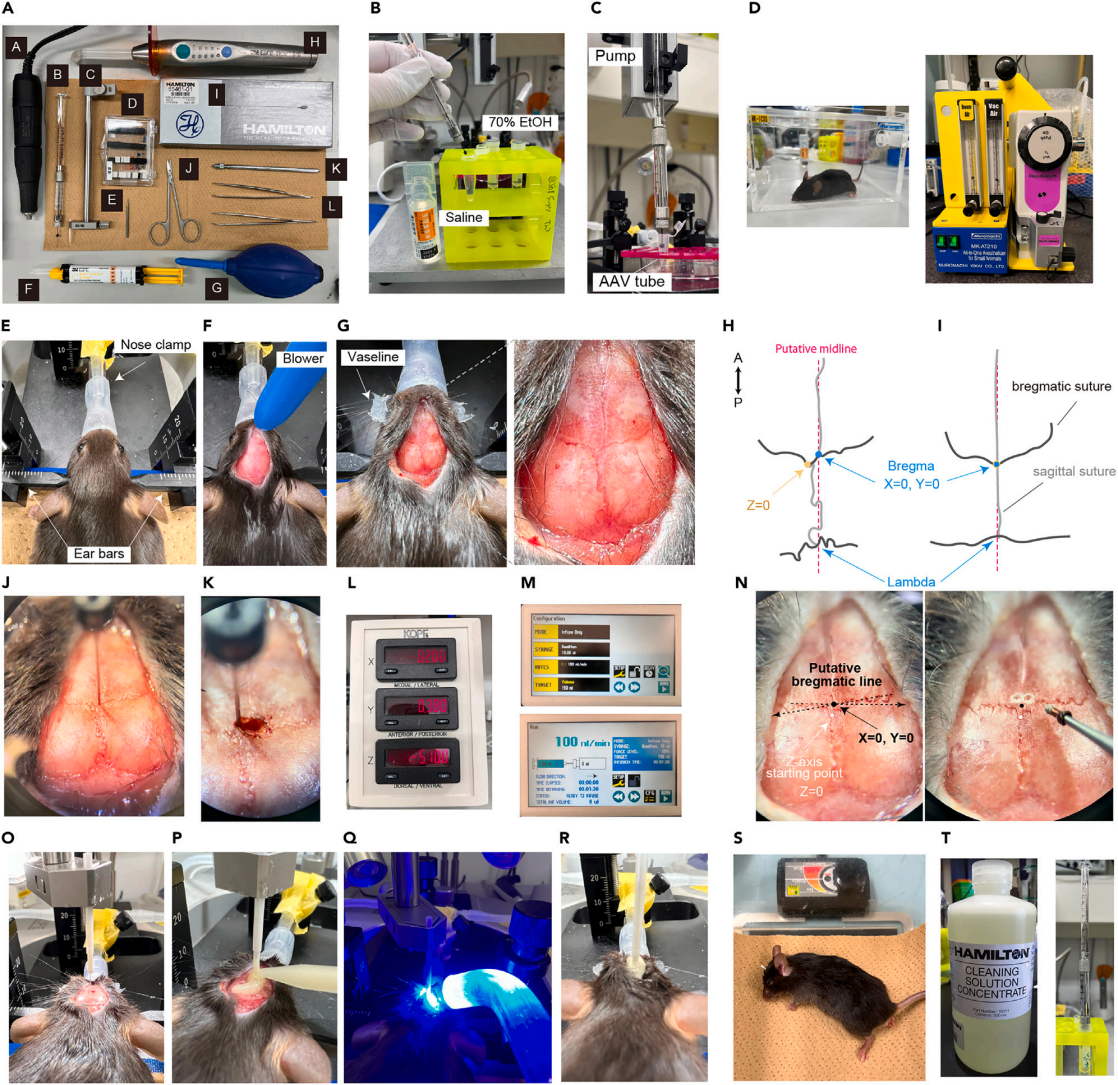
AAV injection and fiber implantation (A) Surgical tools. A- drill. B- Hamilton syringe. C- cannula holder. D- optical fiber. E- drill bit. F- dental cement. G- blower. H- curing light. I- 33G Hamilton needle. J- Scissors. K- tungsten needle. L- tweezers. (B) Wash the Hamilton syringe with 70% EtOH and saline. (C) Load the AAV in the Hamilton syringe. (D) Anesthetize the mouse with 2% isoflurane inhalation. (E) Fix the head of the mouse in the stereotaxic frame. (F) Dry the surface of the mouse skull with a blower. (G) The surface of the mouse skull. (H and I) Schematics showing bregma, lambda, and sutures. An example of bregma (H), in which the sagittal suture is not on the middle line. (I) also shows another example. (J) Stereomicroscopic field of view with needle tip pointing to bregma. (K) Drill a hole in the skull at the target site and insert the syringe. (L) Coordinates of the injection site displayed on a digital board. (M) Configuration for the AAV injection. (N) Bregma and injection sites are shown. The blue point is the bregma (the zero points of the X and Y axes), while the white one is the zero point of Z-axis origin. Right, overhead view of drilling position. (O–R) Implant an optic fiber. Pinpoint the bregma with fiber tip (O). Fix the optical fiber in place using dental cement and curing light (P and Q). Stiff fixation of the fiber should be confirmed (R). (S) Warming the mouse after the surgery until recovery from anesthesia. (T) Cleaning solution for Hamilton syringe and needle.
2. Implantation of optic fibers.
  a. Set a holder of optic cannula (200-μm diameter, NA:0.50, 6.0 mm long) to the stereotaxic mode.
  b. Hold an optic cannula with the holder and set the position of the cannula tip to the bregma (Figure 4O).
  c. Move the cannula to the implantation site and insert it into the brain (+0.38 mm AP, 0.00 mm ML, −4.80 mm DV).
    ***Note:*** Position of cannula tip is slightly above (usually 0.3 mm) the injection site.
  d. Remove fluid (blood, cerebrospinal fluid, etc.) from surface of the skull and fix the implanted optic cannula to the skull using dental cement (Figure 4P).
  e. Expose UV-violet light to the dental cement for 10–20 s by using light-curing type cement (Figure 4Q).
    ***Note:*** Do not look at the light directly.
  f. Apply cement and light irradiation several times to secure the fiber tightly in place (Figure 4R).
    ***Note:*** Screws or other anchors are not necessary.
  g. After the surgery, warm the mice to promote spontaneous recovery (Figure 4S) and carefully wipe off the Vaseline from the eyes.
  h. Once they begin to move spontaneously, place them in individual cages.
  i. Wait for at least three weeks before subsequent experiments to allow for a sufficient recovery period.
    ***Note:*** Once the gene is adequately expressed, QIH can be induced repeatedly with a high reproducibility at least for 8 months after the surgery.
    **CRITICAL:** Because the precise location of the injection and optic cannula implantation is critical for achieving proper manipulation of target neurons, optimize the coordinates of the injection site before conducting experiments. The optimized position may vary depending on factors such as the age of mice and type of stereotaxic frame used.

3. Washing Hamilton syringe and 33G needle.
  a. Wash the needle in the same order as in **step 1b**.
  b. Perform an additional wash using Hamilton™ Needle Cleaning Concentrate, which is diluted to 25% with water, 3 times (Figure 4T).

**CRITICAL:** Clean properly the needle after use as described in **step 1u and 10v**, so that the needle can be reused. It can be used for approximately 10 surgeries (about 40 mice).

### Transmitter implantation into the abdominal cavity

**Timing: 15 min**

**Figure 5:**
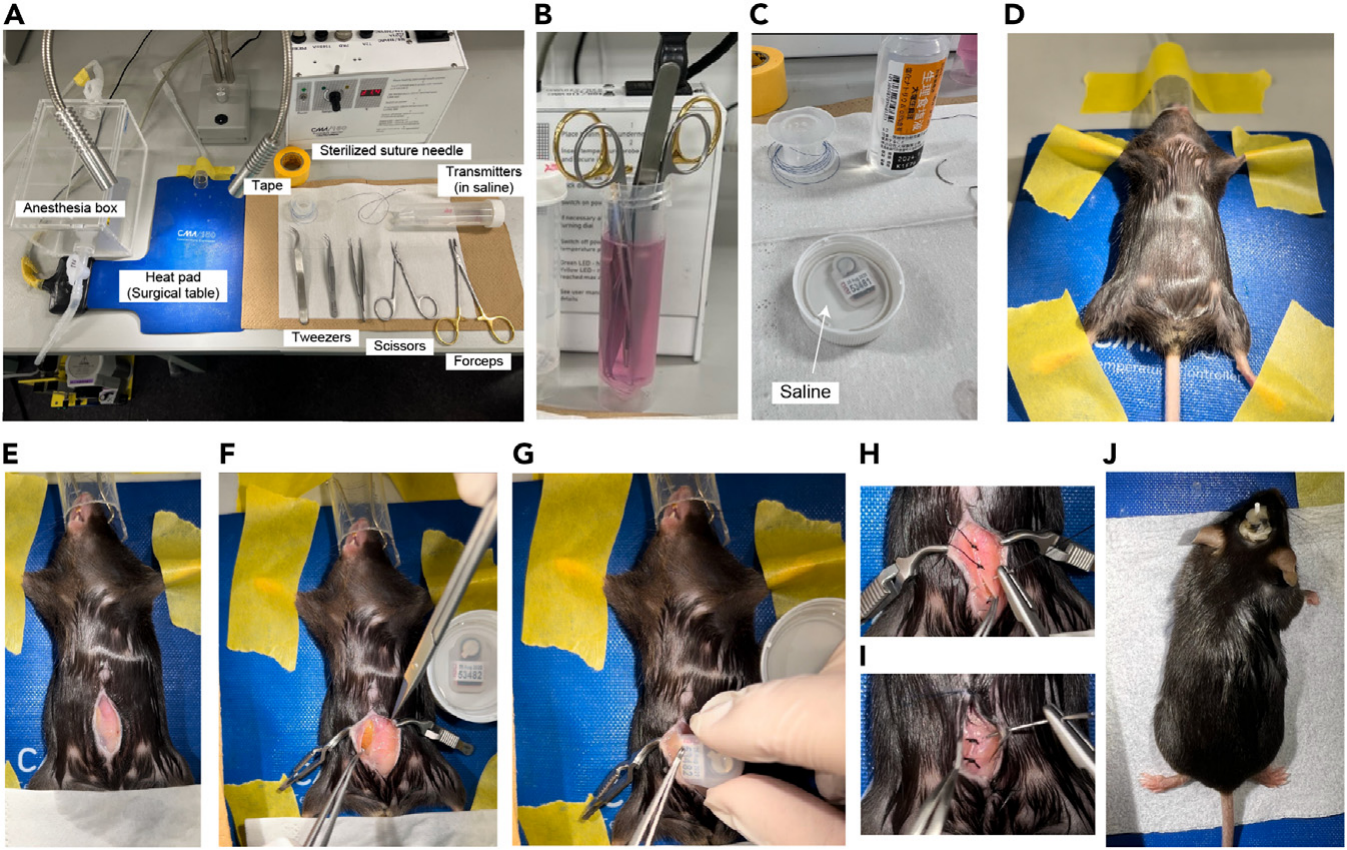
The main experimental steps of transmitter implantation (A) Tools for the implantation. Tape is used to fix the mouse limbs during surgery. (B) Surgical instruments used to cut the peritoneum should be carefully sterilized by an antiseptic solution. (C) Put a transmitter in saline until just before implantation. (D) Place the anesthetized mouse on the heat pad and fix the limbs with tape. (E) Make a 1.5 cm incision in the skin. Clean with saline and keep the tissue wet during the surgery. Eliminate the hair as much as possible. (F) Make a minimal incision in the transparent peritoneum, being careful not to cut the abdominal muscles. (G) Put a transmitter into the abdominal cavity. (H) Suture the peritoneum with sterile thread. (I) Suture the skin with a sterile thread. (J) Wipe the wound thoroughly and warm the mouse after the surgery until it recovers from anesthesia.

This step describes the procedure for implanting the TA-F10 transmitter in the abdominal cavity to monitor the mouse core body temperature (Figure 5). If you intend to monitor only T_BAT_, skip this step.

4. Implantation of transmitters.
  a. Prepare the surgical instruments (Figure 5A).
    **CRITICAL:** Carefully sanitize the instruments used to cut the peritoneum before the surgery.
  b. Put the transmitters in HIBITANE® (diluted to 0.5% with water) in a 50 mL conical tube at least for 10 min (Figure 5B) and wash them with sterilized saline just before the implantation.
  c. Soak a transmitter (TA-F10) in saline until the implantation (Figure 5C).
  d. Anesthetize a *Qrfp-iCre* heterozygous mouse that has received AAV injection and optic cannula implantation using 1–2% isoflurane via ventilation for 5 min.
  e. Position the mouse on its back and fix its limbs to a heat pad using masking tape while maintaining inhalation of isoflurane (Figure 5D).
  f. Thoroughly sanitize the skin around the stomach with 70% ethanol and sterilized saline.
  g. Make a small incision in the middle of the abdomen using scissors (Figure 5E).
    ***Note:*** It is recommended not to shave the hair on the stomachs of mice because shaving may possibly aggravate wounds.
  h. Make a small incision in the peritoneum. An incision of approximately 1 cm is usually sufficient for the TA-F10 implantation (Figure 5F). Methods Video S1.
    **CRITICAL:** Make sure to make an incision in the thin and transparent peritoneum. Avoid cutting the abdominal muscles; otherwise, bleeding may occur. Troubleshooting 4
    Methods Video S1. Transmitter implantation into the abdominal cavity, in reference to step 4h–k in ‘‘Transmitter implantation into the abdominal cavity”
  i. Put a sensor in the abdominal cavity (Figure 5G).
  j. Sew the peritoneum with 3–4 stitches using a sterilized suture needle and suture material (Figure 5H).
    **CRITICAL:** Proceed with this step as quickly as possible to prevent hypothermia in the mice during the operation.
  k. Wash the peritoneum with saline to prevent it from drying out.
    ***Note:*** It is recommended to warm up the saline to approximately 30°C to avoid loss of body temperature.
  l. Sew the skin using a sterilized suture needle and suture (Figure 5I).
  m. Carefully wipe the blood with paper and keep the mouse warm until it moves spontaneously (Figure 5J).
  n. Put the mouse into a warm recovery chamber for the early recovery and transfer the mouse to a clean individual cage and allow it to recover.
    ***Note:*** At least one week is required for the recovery period after the surgery.

### Body temperature recording and optogenetic manipulation

**Timing: hours to several days**

This section describes the procedure for monitoring mouse T_B_ and/or T_BAT_ in free-moving mice subjected to QIH induced by optogenetics. For monitoring either T_B_ or T_BAT_, you should acclimate the mouse to the recording chamber for at least 12 h and record them in a thermostatic chamber (Figures 3A and 3E) to keep the ambient temperature stable. This step also describes optogenetics using OPN4dC. Since OPN4dC is a highly sensitive opsin, blue light exposure does not cause damage to the brain tissues even after stimulation for a long time (at least 24 h).

**Figure 6:**
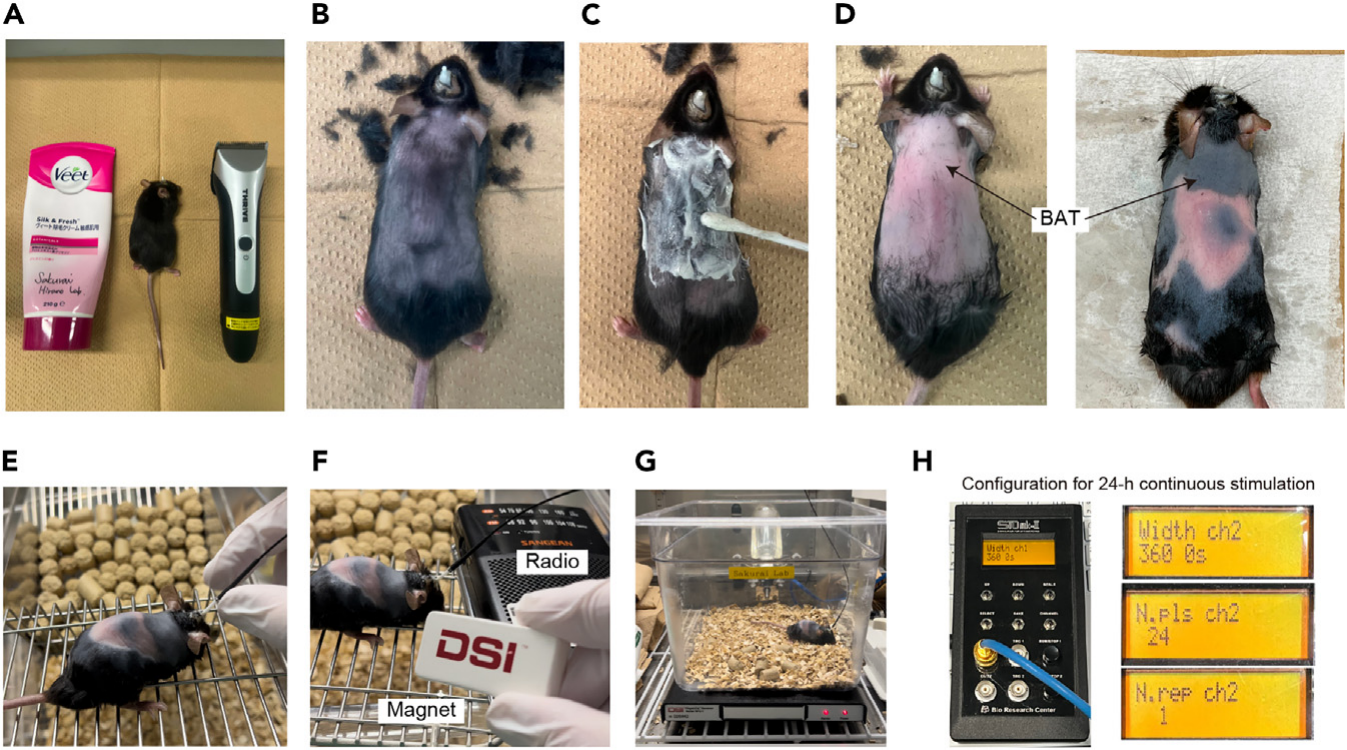
Setup for body temperature recording and photo-stimulation (A) Hair clipper and hair removal cream to expose skin around the interscapular region. (B) Trim back hair closely. (C) Remove the hair with hair removal cream. (D) Examples of mice after removing the hair. (E) Attaching fiber patch cord. (F) Switch on the transmitter by the magnet and confirm by radio. (G) Put the mouse in the recording cage. (H) The setting of the pulse generator. For 24 h of continuous stimulation, the system is designed to deliver 24-pulse with a pulse width of 3600 s without interval.

**Figure 7:**
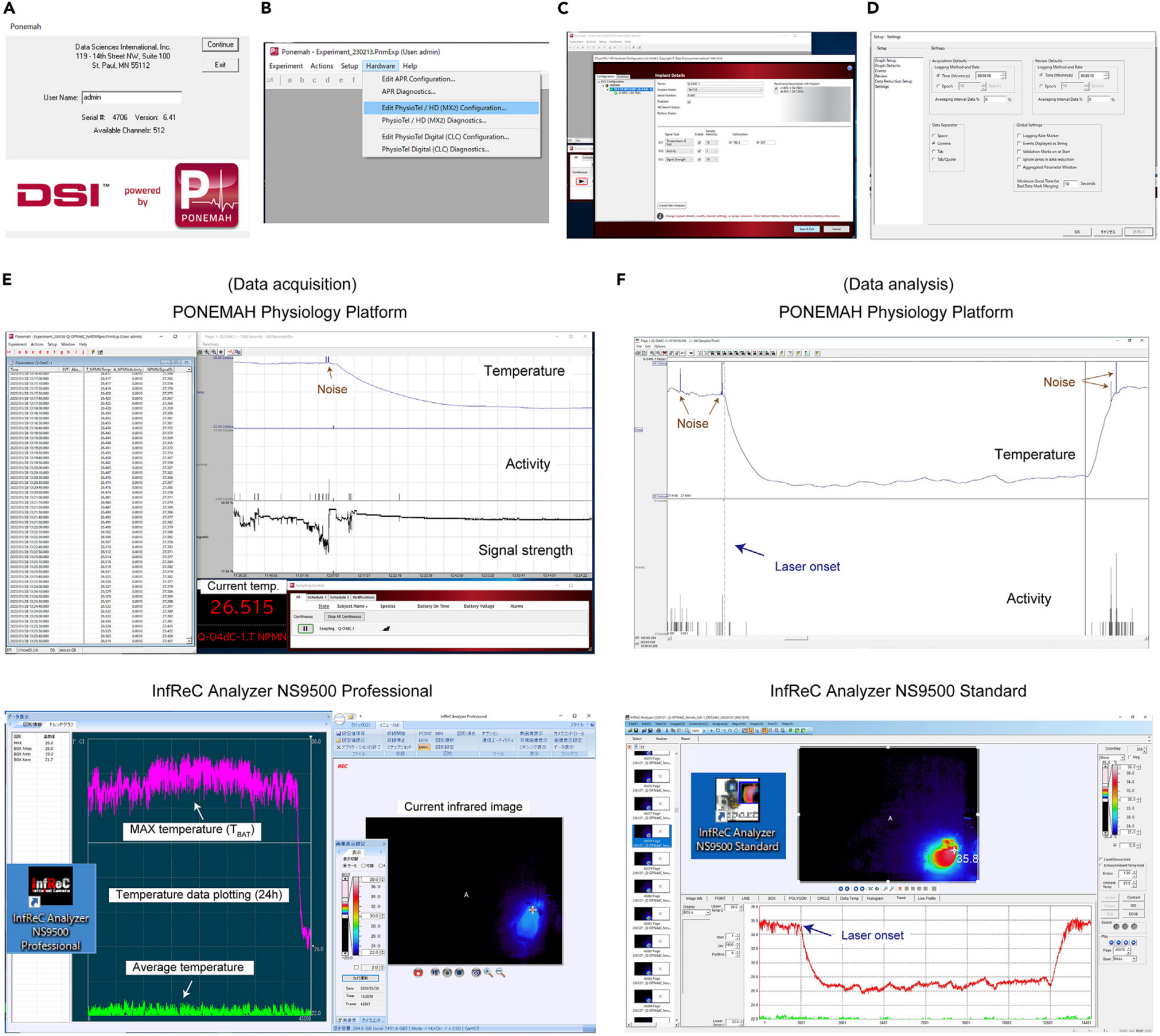
Recording of the core and surface body temperatures (A) First screen when Ponemah software is opened. (B) The setting of the hardware. (C) Registration of matrix, transmitter, and receiver. (D) Setup the experiment schedule. (E) Live view of data collection using telemetry (Ponemah, upper) and infrared camera (InfRec, lower). Both photographs were taken 1.5 h after the start of laser stimulation. (F) Raw data obtained using telemetry (Ponemah, upper) and infrared camera (InfRec, lower). The body temperature for 1-h pre-stimulation, 6-h stimulation and 1-h post-stimulation is shown.
  b. Put an RPC-1 receiver board (Figure 3D) under the mouse cage and connect the board to the matrix with a LAN cable (Figure 3C).
  c. Edit PhysioTel/HD(MX2) configuration (Figure 7B).
  d. Register the “Hardware” (registration of transmitter TA-F10 and receiver RPC-1) (Figure 7C), according to the PONEMAH manual.
  e. Register the “Experiment Setup” and “Subject Setup” (Figure 7D).
  f. Turn on the power of the transmitter embedded in the mouse with a magnet (same as in Figure 6F).
  g. Use a radio (AM52) to check whether the transmitter is actually on.
  h. Start acquiring data and confirm that the value of the body temperature detected by the TA-F10 is stable (Figure 7E). Troubleshooting 6.
    ***Note:*** TA-F10 can measure the mouse locomotor activity as well as temperature.
  i. Record parameters at desired acquisition rate.
    ***Note:*** The sampling rate at 0.1 Hz (once in 10 s) is sufficient, but it depends on the grade of the matrix and the number of mice.

5. To precisely detect the T_BAT_ and surface temperature of mice, remove the hair around the interscapular region before the experiment (Figures 6A–6D).
  a. Prepare a hair clipper and hair removal cream (Figure 6A).
  b. Anesthetize a mouse with ventilated 1–2% isoflurane for 2 min.
  c. Cut the hair with the clipper and apply hair removal cream on the back (Figure 6B).
  d. Spread it thoroughly with a cotton swab.
  e. Anesthetize the mouse again and wait for 1 min (Figure 6C).
    ***Note:*** Do not leave the cream on for more than one minute.
  f. Remove the cream carefully with a piece of paper moistened with warm water (Figure 6D).
    ***Note:*** Even when the hairs on the back are not incompletely removed since the hair follicles are often left behind (Figure 6D, right), it does not cause problems in experiments that end within a week.
    **CRITICAL:** Remove hair on the back of mice just before the T_BAT_ measurement, since the hair, especially of young mice, grows in a few days.

6. Setting up of infrared sensor camera for T_BAT_ measurement and optogenetics.
  a. Start InfReC software (InfReC Analyzer NS9500 Professional, Nippon Avionics) used to obtain and analyze images.
  b. Turn on the infrared thermographic camera at least 1 h before use for calibration.
    ***Note:*** To maximize the accuracy of measurements, keep the camera in a thermostatic chamber at all times (Figure 3E).
  c. Confirm that the cage temperature detected by the camera is the same as the ambient temperature and stable.
  d. Adjust the power of the laser to an ideal range of 3–10 μW at the fiber tip (Figure 3I).
    ***Note:*** The 3–10 μW laser is very weak, but it is visible in the dark.
    **CRITICAL:** Provide the light to neurons continuously (not pulsed).
  e. Connect the optical fiber implanted in the mouse brain to the fiber patch cord (200-μm diameter, NA:0.57, 1m in our study) at least 1 h before the manipulation for the mouse to get habituated to it (Figure 6E). Troubleshooting 5.
    ***Note:*** If you are also using the telemetry system, turn on a transmitter embedded in the mouse with a magnet (Figure 6F), as described below (**step 7**).
  f. Put a mouse in a cage, above which the infrared camera is set (Figure 6G).
  g. Ensure that the rotary joint allows the mouse to move freely.
  h. Confirm that the camera detects the temperature around the interscapular region, which should show the highest value in the mouse. The usual temperature of T_BAT_ in mice is 35°C–38°C
  i. Record infrared thermographic images at the desired acquisition rate.
    ***Note:*** The sampling rate of 0.5 Hz (1 frame per 2 seconds) is enough to track the mouse movements as well as changes in the body temperature.

7. Setup PhysioTel telemetry system for T_B_ measurement.
  a. Start up the PhysioTel system and open the software (PONEMAH Physiology Platform v.6.30) (Figure 7A).

8. Optogenetic manipulation.
  a. Set the stimulation condition with TTL pulse generator (Figure 6H).
  b. Apply the blue light stimulation continuously.
9. Data analysis of T_BAT_ and T_B_.
  a. Monitor the body temperatures (Figure 7E). Troubleshooting 7.
    ***Note:*** In the acquisition mode, thermal images show the highest value within the recording area. The highest value usually indicates the mouse BAT temperature.
  b. Once data acquisition by the thermal camera is completed and data is saved, you can analyze data using official InfReC software (InfReC Analyzer NS9500 Standard, Nippon Avionics) (Figure 7F, Methods Videos S2, S3, and S4). On the other hand, in the PhysioTel telemetry system, you can perform data analysis during the data acquisition itself using PhysioTel (PONEMAH Physiology Platform v.6.30).
    Methods Video S2. InfraRed thermal image movie for a total of three days (January 29, 2023, at 14:00 - February 1, 2023, at 14:00) before and after 24-hour stimulation (10 μW), with reference to “Expected outcomes” Photo stimulation started on January 30, 2023, at 14:00 (ZT=6). The video was recorded at 3600x speed. The video was divided into three parts: first (S2), middle(S3), and final third(S4).2
    Methods Video S3. InfraRed thermal image movie for a total of three days (January 29, 2023, at 14:00 - February 1, 2023, at 14:00) before and after 24-hour stimulation (10 μW), with reference to “Expected outcomes” Photo stimulation started on January 30, 2023, at 14:00 (ZT=6). The video was recorded at 3600x speed. The video was divided into three parts: first (S2), middle(S3), and final third(S4).3
    Methods Video S4. InfraRed thermal image movie for a total of three days (January 29, 2023, at 14:00 - February 1, 2023, at 14:00) before and after 24-hour stimulation (10 μW), with reference to “Expected outcomes” Photo stimulation started on January 30, 2023, at 14:00 (ZT=6). The video was recorded at 3600x speed. The video was divided into three parts: first (S2), middle(S3), and final third(S4).4

## Expected outcomes

Without stimulation (baseline), the body temperature of mice is basically 36–38°C in the dark period and 35–36°C in the light period. To confirm the reduction in body temperature during the photo-stimulation, either surface body temperature (T_BAT_) and core body temperature (T_B_) can be used, while there can be a few differences between T_BAT_ and T_B_.

**Figure 8:**
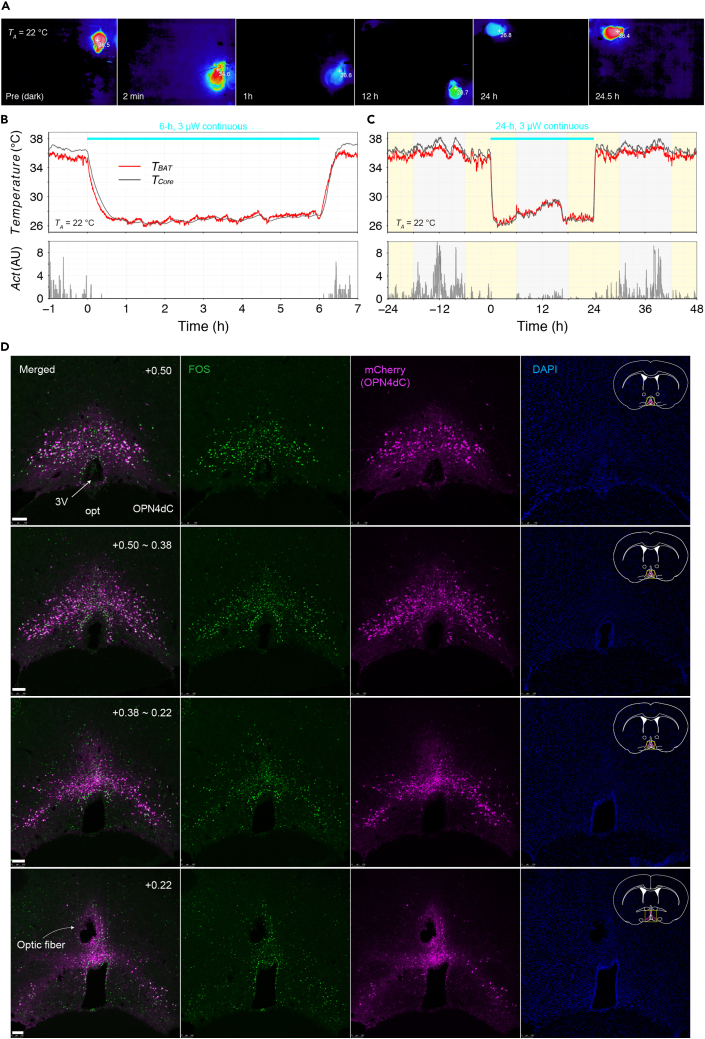
Representative data of long-term recordings of the body temperature (A) Representative thermography images. These images were picked up from Methods Video S2, S3 and S4. (B and C) The body temperatures and activity during 6-h (B) and 24-h (C) stimulation (3 μW continuous light) were plotted. Both data were obtained from the same mouse. Yellow and gray boxes in (C) show light and dark phases, respectively. (D) Brain slice validating OPN4dC expression in Q neurons and the position of optic fiber. Representative coronal brain sections (AP +0.50 ∼ +0.22 mm from bregma) of mice used in Figures 8A–8C show the expression of OPN4dC-mCherry (magenta) and c-Fos (green) 3 h after the optic stimulation. 3V; third ventricle, opt; optic tract. Scale bars; 100 μm.

After the initiation of photostimulation, tail vasodilation should be observed within 2 min, which can be detected by the thermographic camera (Figure 8A), as shown in Methods Video S3. When the ambient temperature is 22°C, the mouse body temperature should decrease to approximately 26–27°C within an hour and remain at a low level during the manipulation (Figures 8A–8C). Once the manipulation is terminated, the body temperature should quickly increase and return to the normal level within 30 min (Figure 8). Detailed changes in physiological parameters (body temperature, activity, heart rate etc.) are shown in Takahashi et al. (2022).^1^ The mouse used for Figure 8 had been subjected to viral injection more than 8 months prior to the recording, indicating the stable expression of AAV and induction of QIH.

***Note:*** Body temperature data from thermographic cameras often show low values when mice are moving vigorously. To exclude as much of the artificial effect as possible, we use the highest value every 5 frames as sample data. In this case, when the sampling rate is 0.5 Hz, the body temperature should be plotted every 10 seconds.

## Limitations

We have previously reported that OPN4dC-inducible QIH lasts for at least 24-h and that 24-h QIH can be repeatedly induced at least 4 times using the same mouse without any observed brain damage.^1^ However, it is not guaranteed that this will be the case for more frequent or prolonged induction of QIH. It is possible that the efficiency of QIH induction may be reduced due to brain damage or bleaching of the OPN4dC.

The body temperature during QIH is partially affected by the circadian clock and raises to 28–29°C during the dark period probably due to the increase of the mouse locomotor activity.

## Troubleshooting

### Problem 1

Mice died during stereotaxic surgery. Related to **step 1** in ‘‘AAV injection and optic fiber implantation’’.

### Potential solution

There are some possibilities; 1) The ear bars are closed too tightly. 2) The temperature of the heat pad has been set too high. High temperature is harmful, especially to small animals such as mice. 3) The concentration of the isoflurane is too high. The breathing of the mice should be checked constantly.

### Problem 2

Strange sutures in the skull make it difficult to position bregma. Related to **step 1k** in ‘‘AAV injection and optic fiber implantation’’.

### Potential solution

The bregma should be at the intersection of the bregmatic suture and sagittal suture, but the sagittal suture could not be found on the center line (as shown in Figures 4G and 4H) or intersection posteriorly wedged (Figure 4N). In the former case, the midline is estimated based on the position of mouse eyes and the lambda and the bregma is determined at the intersection of the estimated line and the bregmatic suture (Figure 4H). The zero point of Z-axis is the intersection of the actual bregmatic suture and sagittal suture. In the latter case, the bregmatic suture should be extended as shown in the Figure 4N to determine the bregma.

### Problem 3

The expression of OPN4dC-mCherry is weak. Related to **step 1t** in ‘‘AAV injection and optic fiber implantation’’.

### Potential solution

Try more concentrated virus vectors and optimize the injection site. Home-made virus vectors without ultra-centrifuge purification should be fine for QIH induction.

### Problem 4

Accidentally cut the abdominal muscles of a mouse, causing bleeding. Related to **step 4**h in ‘‘Transmitter implantation into abdominal cavity’’.

### Potential solution

Immediately stop making the incision, swab the blood, and let it heal naturally. If the wound is large, it should be stitched. Damage to the abdominal muscles will most likely diminish the activity of a mouse. An incision should be made in a thin, transparent part of the peritoneum.

### Problem 5

The patch cord is damaged by being bitten during recording. Related to **step 6e** in ‘‘Body temperature recording with optogenetic manipulation’’

### Potential solution

The length of the patch cord and distance between the mouse cage and the rotary joints should be optimized not to be touched by mice. See also “preparation for optogenetic manipulation”.

### Problem 6

Noise is observed in telemetry data. Related to **step 7h** in ‘‘Body temperature recording with optogenetic manipulation’’

### Potential solution

Slight noise is usually observed (Figures 7E and 7F), when mice are highly active. However, when receivers are close to each other (approximately less than 30 cm distance between the two) or when there is another transmitter near by the receiver, it causes a strong noise. The noise also occurs when transmitter batteries are about to run out. These noisy data can be automatically excluded using the PONEMAH analysis software.

### Problem 7

Temperature does not decrease after the photo-stimulation. Related to step 9a in ‘‘Body temperature recording with optogenetic manipulation’’

### Potential solution

First, confirm the intensity of light and wavelength for stimulation. Strong blue light (<100 μW from fiber tip) possibly decreases the efficiency of manipulation. 3 μW–10 μW power at the fiber tip is ideal for OPN4dC (Figure 3I, left). Second, confirm the expression of OPN4dC-mCherry at the AVPe in the brain (Figure 8D)

## Resource availability

### Lead contact

Requests for further information, resources and reagents should be directed to the lead contact, Tohru M. Takahashi (tmtakahas@gmail.com).

### Materials availability

This study did not generate new unique reagents.

## Data Availability

The published article includes all the data generated during this study.
